# Cell cycle related proteins in hyperplasia of usual type in breast specimens of patients with and without breast cancer

**DOI:** 10.1186/1471-2121-7-29

**Published:** 2006-07-26

**Authors:** Luciene SA Tafuri, Gislene FS Rocha, Helenice Gobbi

**Affiliations:** 1 Department of Anatomic Pathology, School of Medicine, Federal University of Minas Gerais, Av. Alfredo Balena, 190, Belo Horizonte, Minas Gerais, 30150-270, Brazil

## Abstract

**Background:**

Hyperplasia of usual type (HUT) is a common proliferative lesion associated with a slight elevated risk for subsequent development of breast cancer. Cell cycle-related proteins would be helpful to determine the putative role of these markers in the process of mammary carcinogenesis. The aim of this study was to analyze the expression of cell cycle related proteins in HUT of breast specimens of patients with and without breast cancer, and compare this expression with areas of invasive carcinomas.

**Results:**

Immunohistochemical evaluation was performed using antibodies against cell cycle related proteins ER, PR, p53, p21, p63, and Ki-67 in hyperplasia of usual type (HUT) in specimens of aesthetic reduction mammaplasty (ARM), in specimens of mammaplasty contralateral to breast cancer (MCC), and in specimens of invasive mammary carcinomas (IMC) presenting HUT in the adjacent parenchyma. The results showed that the immunoexpression of ER, PR, p21, p53, p63, and KI-67 was similar in HUT from the three different groups. The p63 expression in myoepithelial cells showed discontinuous pattern in the majority of HUT, different from continuous expression in normal lobules. Nuclear expression of p53 and p21 was frequently higher expressed in IMC and very rare in HUT. We also found cytoplasmic expression of p21 in benign hyperplastic lesions and in neoplastic cells of IMC.

**Conclusion:**

Our data failed to demonstrate different expression of cell cycle related proteins in HUT from patients with and without breast cancer. However, we found discontinuous expression of p63 in myoepithelial cells around HUT adjacent to carcinomas and cytoplasmic expression of p21 in epithelial cells of hyperplastic foci. Further studies are needed to determine how these subgroups relate to molecular abnormalities and cancer risk.

## Background

The hypothetical multistep model of carcinogenesis indicates that breast cancer develops via a series of intermediate hyperplastic lesions through in situ to invasive carcinomas, with the risk of developing carcinoma increasing at each stage [1-3]. Epidemiological studies demonstrated an increased risk of developing breast cancer associated with proliferative breast lesions. Hyperplasia of usual type (HUT) is a common proliferative lesion associated with a slightly elevated risk for subsequent development of breast cancer (relative risk = 1.6, augmented to 2.1 with positive family history) [1,2,4]. HUT is not necessarily a direct precursor of invasive breast carcinoma but may identify individuals whose breast tissue has acquired a molecular alteration that can facilitate the eventual development of this disease [5].

The defective function of regulatory cell cycle elements, like estrogen and progesterone receptors, Ki-67, p53, p21^WAF1 ^and p63 leads toward increased proliferation and, in addition, expansion of genome damaged cells. Cell cycle-related markers would be helpful to determine the putative role of these markers in the process of mammary carcinogenesis [6].

Many studies evaluated cell cycle-related proteins in invasive breast carcinomas, but there are few studies evaluating these proteins in HUT [7-9]. Some molecular alterations may already be present in the earliest stages of breast cancer development. Detection of these alterations may be important for understanding the pathogenesis and also for risk assessment of premalignant breast lesions.

Incidental cancers or precursor lesions are rare in specimens of cosmetic mammaplasty compared to reduction mammaplasty specimens performed for symmetry of contralateral breast in women with breast cancer undergoing mastectomy or conservative surgery. Our hypothesis is that the expression of cell cycle related proteins would be different in HUT lesions from women at higher risk of breast cancer or with breast cancer, compared to those HUT from women without breast cancer. The aim of this study is to analyze the expression pattern of cell cycle related proteins ER, PR, p53, p21^WAF1^, p63, and Ki-67 in hyperplasia of usual type (HUT) of breast specimens of patients with and without breast cancer, and compare this expression with neoplastic cells of invasive carcinomas.

## Results

The age of patients submitted to ARM ranged from 30 to 67 years (mean 43.9 years; SD = ± 7.4 years), of patients with IMC ranged from 30 to 86 years (mean 55. 7; SD = ± 13.1 years), and age of patients submitted to MCC ranged from 30 to 75 years (mean 51.6; SD = ± 12.4 years). Patients were divided according to the menopausal status into pre-menopausal patients (≤ 50 years) and post-menopausal patients (> 50 years). The mean age of patients with IMC and submitted to MCC was significantly greater (Table 2) than patients submitted to ARM (p < 0.005). There was no statistically significant difference between mean age of patients from IMC and MCC groups.

**Table 1:** Primary antibodies, dilutions, and sources of antibodies used in immunohistochemical study

***Antibody***	***Clone***	***Dilution***	***Pretreatment***	***Source/Country***
ER	6F-11	1:100	Steamear/citrate	Novocastra/UK
PR	PgR 312	1:100	Steamear/citrate	Novocastra/UK
p53	DO-7	1:500	Steamear/citrate	Dakocytomation/USA
p21	4D10	1:20	Steamear/citrate	Novocastra/UK
p63	4A4	1:100	Steamear/EDTA	Dakocytomation/USA
Ki-67	MIB-1	1:100	Steamear/citrate	Immunotech/France

**Table 2:** Menopausal status in patients submitted to aesthetic reduction mammaplasty (ARM), to mammaplasty contralateral to breast cancer (MCC), and patients with invasive mammary carcinoma (IMC)

***Menopausal status***	***ARM***	***MCC***	***IMC***
< 50 years	30 (88.2%)	7 (46.7%)	13 (38.2%)
≥ 50 years	4 (11.8%)	8 (53.3%)	21 (61.8%)

Total	34 (100%)	15 (100%)	34 (100%)

**Figure 1:**
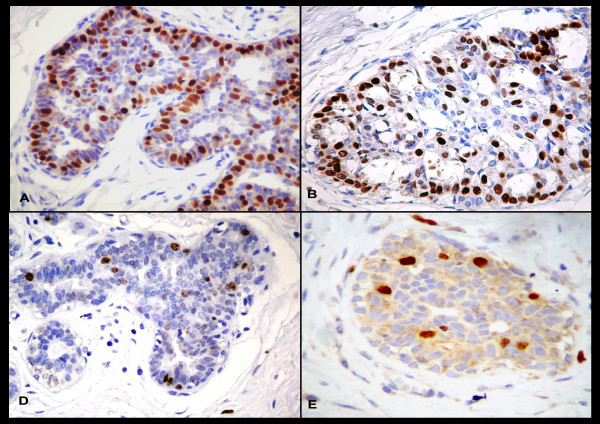
(A) Positive immunostaining for ER in nuclei of epithelial cells of HUT (400×); (B) Positive immunostaining for PR in nuclei of epithelial cells of HUT (200×); (C) Positive immunostaining for p21 in nuclei of epithelial cells of HUT (400×); (D) Positive immunostaining for Ki-67in nuclei of epithelial cells of HUT (400×).

The histologic review showed that HUT was associated with other benign breast lesions in the majority of the cases. Histologic findings were varied in ARM, MCC, and IMC specimens. Usually, the strongest ER staining was noted at the periphery of the hyperplastic foci (Figure 1A). The majority of the epithelial cells of HUT in all specimens showed positive staining for ER, PR, and Ki-67 (Figure 1; Table 3). The ER immunostaining was localized in the nuclei and showed some variability in intensity even in individual lesions of the same case.

**Table 3:** Positivity for ER, PR, p53, p21, p63, and KI-67 in HUT areas of reduction aesthetic mammaplasty specimens (HUT-ARM), HUT of mammaplasty contralateral to breast cancer (HUT-MCC); HUT adjacent to invasive mammary carcinoma (HUT-IMC), and in invasive mammary carcinoma (IMC)

***Antibody***	*HUT-ARM n (%)*	*HUT-MCC n (%)*	*HUT-IMC n (%)*	*IMC n (%)*
RE	33 (97.1)	13 (86.7)	32 (94.1)	18 (52.9)
RP	34 (100)	12 (80,0)	33 (97.1)	18 (52.9)
p53	0 (0)	0 (0)	0 (0)	19 (55.9)
p21	0 (0)	0 (0)	N*- 2 (5.9)	N*- 19 (55.9)
			C*- 6 (17.6)	C*- 8 (23.5)
p63	34 (100)	15 (100)	34 (100)	3 (8.8)
Ki-67 **	L- 20 (58.8)	L- 4 (26.7)	L- 22 (64.7)	L- 9 (26.5)
	I- 2 (5.9)	I- 2 (13.3)	I- 5 (14.7)	I- 17 (50)
				H- 8 (23.5)

* Localization of p21 staining: N- nuclear, C- cytoplasmatic

** KI-67 expressed as: L-low, I-intermediate, and H-high proliferative index

**Figure 2:**
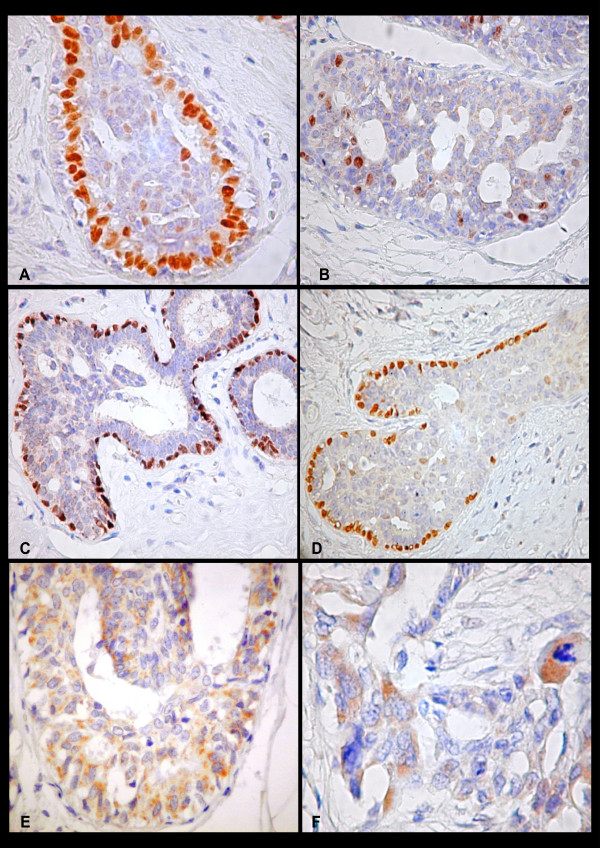
Positive immunostaining for ER (400×) (A) and for Ki-67 (400×) (B) in nuclei of epithelial cells of HUT, note in both the marked staining of the most of the cells at the periphery of the spaces. Positive immunostaining for p63 in nuclei of myoepithelial cells of HUT occurred as a continuous layer (C) (200×); and occurred as a discontinuous layer pattern (200×)(D)in nuclei of myoepithelial cells of HUT. Positive immunostaining for p21in cytoplasm of epithelial cells of HUT (400×) (E) and in invasive mammary carcinoma (400×) (F).

The p63 expression was detected in the majority of the myoepithelial cell nuclei in normal lobules and in HUT. p63-positive cells around HUT foci occurred as a discontinuous layer in 38.1% in ARM, in 73.3% in MCC, and in 64.7% of myoepithelial cells surrounding HUT adjacent to IMC (Figure 2C and 2D). The p63 expression was continuous in myoepithelial cells of normal lobules and ducts. There was no difference in the percentage of positive cells for ER, PR, p21^WAF1^, p53, p63, and KI-67 in HUT of ARM, MCC and IMC (p > 0, 05). The mean percentage of ER+, PR+, Ki-67+ in epithelial cells and, p63+ in myoepithelial cells of HUT from all groups was significantly higher than positivity in neoplastic cell of IMC (Table 3).

Usually, neoplastic cells of IMC showed intermediate to high proliferative index of Ki-67 positivity (73.5%) compared to positive cells of HUT from ARM specimens (5.9%), MCC specimens (13.3%) and HUT-IMC (14.7%), (Table 3). No significant difference was observed between Ki-67 expression in cells of HUT-ARM and HUT-IMC. All proliferative cells of HUT of the three different groups were negative for p53.

The p21 expression in IMC was predominantly nuclear (55.9%). Cytoplasmatic staining was seen in neoplastic cells in 23.5% of cases (Figure 2F). Nuclear staining was detected in cells of HUT-MCC in 2 cases (5.9%; Figure 1D) and 6 cases showed cytoplasmatic staining in hyperplastic cells (Figure 2E).

## Discussion

The aim of this study was to determine alterations in the expression of proteins involved in proliferation and cell cycle in HUT cells of patients with and without invasive breast cancer. Our analysis showed no difference in the cell cycle related proteins immunoexpression in HUT from the three different groups, in spite of age and menopausal status. Similar results were obtained by Gobbi *et al*. (2005)[7] evaluating ER expression in usual hyperplasia without atypia of patients who developed breast cancer compared with patients who did not.

In our study, we found that ER and PR immunoexpression was significantly higher in HUT cells than in neoplastic cells of IMC specimens. Even in all 16 cases of ER-negative tumors the epithelial cells of HUT were positive for ER. Our results are in agreement with other investigators who found higher levels of ER expression in benign breast epithelium of patients who developed breast cancer compared to controls [7,9-11]. The presence of positive ER staining in normal lobules increases the breast cancer risk and the likelihood of progression to cancer [7]. It occurs through the increase of the rate of cell proliferation by both recruiting non-cycling cells into the cell cycle and by shortening the overall cell cycle time due to a reduction in the length of G1 phase [11].

Previous comparison between normal and precancerous breast biopsies has shown that ER expression is relatively low in normal epithelium and slightly increased in HUT [12]. Recent studies indicate that HUT is a heterogeneous entity containing subgroups identified according to the criterion of ER-α (+) proliferating cells and this fact could explain the different biologic behavior of HUT [13]. In our study, the ER positive cells were more often found at the periphery of hyperplastic lesions. Similar pattern of ER positivity was previously described by Gobbi et al(2005)[7]. It is possible that the ER+ epithelial cells at the periphery of HUT represent the most proliferative group of cells, in spite of low positivity for Ki-67 in sequential sections of the same lesion. The estrogen exposure may stimulate a clonal proliferation of some ER+ cells or may increase the chance of spontaneous mutations [9].

In normal breast, there is a negative association between expression of ER and Ki-67, indicating either that ER+ cells are non-dividing or that the receptor is down-regulated as cells enter cycle [12,14]. This important correlation breaks down in many ER+ cancers, where the receptor is often detected in proliferating cells [14]. However, cells co-expressing ER-α and Ki-67 have been found in precancerous lesions and correlate positively with the level of risk of developing breast cancer [11]. In our series, we found higher ER and Ki-67 immunoexpression in HUT areas compared to the immunopositivity for these markers in adjacent normal lobules. Our results are similar to those described by Schmitt (1995) [11] who found the existence of a positive correlation between ER status and proliferation in hyperplasic epithelium and a progressive inversion of this relationship in lesions evolving towards malignancy. The observation of higher rates of proliferation in ER positive benign proliferative breast lesions fits with the concept of an initial hormone-dependent status in breast carcinogenesis [11]. In addition, HUT with higher expression of ER and Ki-67 could represent a subset of hyperplastic lesions with increased risk of subsequent breast cancer development.

Some previous studies suggest that at least some HUTs are clonal [9,15]. Nevertheless, it remains unclear whether the dysregulation of ER has arisen prior to clonal expansion of the HUT, since cells with apparently abnormal regulation of ER during cell division are scattered randomly throughout the HUT in a non-contiguous pattern in some cases. The variable number of ER+ cells might indicate that in HUT the dysregulation of ER expression is incomplete and may be absent in some lesions, or apparent under certain conditions [13].

We detected p53 and p21 positivity in neoplastic cells of IMC, especially in high grade carcinomas. Mutations in tumor suppressor gene TP53, which mediates G1 arrest and apoptosis leads to an increased half-life and accumulation of the p53 protein [16]. A way to investigate the functional status of TP53 is to evaluate some of its downstream effectors such as p21 gene whose product acts by blocking cyclin-dependent kinases [17]. In our study, we found a positive association between expression of p21 and p53 in neoplastic cells, which is in agreement with the studies of Bankfalvi et al (2000)[18] and Pelikainen et al (2003)[19].

High expression of p21 would result in decreased cell proliferation subsequent to inhibition of cyclin/CDK activity [20]. However, in our study p21 expression in neoplastic cells was related to higher proliferative index. Our results are in agreement with the theory of p53 independent pathways of p21 regulation in breast cancer [21,22]. High amounts of p21 in high proliferating cells may reflect an unsuccessful effort to halt proliferation. This may result from the presence of other cell cycle regulatory pathways, which bypass the p21 mediated cell cycle block, such as c-Myc or B-myb [17] or due to mutant non-functional forms of p21 which posses prolonged half lives [23]. In addition, p21 expression can indeed be up-regulated by epidermal growth factor receptor and transforming growth factor β1 [17] which are associated with higher tumor grade and disease progression in breast carcinoma [24,25].

In our study, cytoplasmic expression of p21 was found in 17.6% of benign hyperplastic cells and in 23.5% of neoplastic cells of IMC. Previous studies have reported exclusive nuclear localization of p21 in neoplastic cells of breast carcinomas [19,23], and in epithelial cells of HUT [8] However, other authors reported p21 immunoexpression in the cytoplasm of breast and ovarian tumors and it was considered critical for promoting cell transformation [26,27]. There is no other data in current literature concerning cytoplasmatic p21 expression in HUT similar to our findings. It remains unclear how the elevated cytoplasmic p21 expression might contribute to tumorigenesis. One possibility is that p21 is sequestered away from the nucleus thereby preventing it from binding to nuclear cyclin/CDK complexes, thus allowing sufficient cyclin/CDK activity for cell cycle progression [28]. Alternately, relocalization of p21 to the cytoplasm may target cytoplasmic molecules such as apoptosis signal-regulating kinase 1 (ASK1) thereby promoting cell survival [29].

In our study, p63 was exclusively expressed in myoepithelial cells of normal breast lobules and ducts, partially expressed around the HUT cells, and rarely expressed in invasive breast carcinoma. We observed that p63 staining was discontinuous in 38.1% in HUT-ARM, in 73.3% in HUT-MCC, and in 64.7% in HUT-IMC. The discontinuous p63 expression pattern in HUT was different from continuous expression in normal lobules and could suggest that there is loss of p63 expression in the progression to invasive carcinoma. Our data is similar to the findings of Wang et al (2002)[30] that demonstrate non-continuous expression of p63 in usual ductal hyperplasia. P63 expression has been useful to differentiate DCIS from microinvasive and invasive carcinomas based on lack of myoepithelial cells in invasive tumors without continuous distribution [31-33]. Although p63 is the most specific marker for myoepithelial cells, limitations exist because discontinuous myoepithelial layer seen in benign lesions, such as in our study may potentially cause diagnostic problems in clinical practice [34].

Although our data and genetic studies failed to demonstrate molecular changes in HUT, that are present in columnar cell lesions, ADH cells and in neoplastic cells of DCIS and IMC [36] the argument that HUT may be an early precursor is still supported by consistent data from epidemiological studies [1-4,37,38].

## Conclusion

Our findings and previously published data [36,37] demonstrate that the imunoprofile of HUT is different from other accepted precursor lesions, since they are composed of a mixed population of cells types with variable proportions of cell-cycle related protein expression, and some alterations could be present in the latest stages of breast cancer development.

## Methods

We selected slides and formalin-fixed, paraffin-embedded blocks from 83 female mammary specimens examined in the Breast Pathology Laboratory of Hospital das Clínicas of Federal University of Minas Gerais received from 1996 to 2004. The specimens selected were 34 specimens of aesthetic reduction mammaplasty (ARM), 15 specimens of mammaplasty contralateral to breast cancer (MCC), and 34 specimens of invasive mammary carcinomas (IMC) presenting HUT in the adjacent parenchyma. The aesthetic reduction mammaplasty was indicated only for cosmetic reasons or for back pain related to hypertrophic breast. There was no clinic or mammography alteration in the breasts of these patients. The mammaplasties contralateral to breast cancer were indicated in order to obtain an aesthetic balance and equilibrium related to the contralateral lumpectomy or mastectomy indicated because of breast cancer. Clinical and pathological data were obtained from the Breast Pathology Laboratory and hospital files. Clinical features evaluated were age, and menopausal status. Slides were reviewed by two observers and criteria used to classify HUT were those from Page & Anderson (1987)[39] and the terminology adopted by the WHO classification [40]. We performed immunostainings using monoclonal antibodies (summarized in Table 1) and the streptavidin-biotin method (Biogenex, USA) with previous heat-induced epitope retrieval. Immunoreactivity for ER, PR, p53, p21^WAF1^, p63, and Ki-67 was evaluated in HUT of ARM specimens (HUT-ARM), in HUT of MCC specimens (HUT-MCC), in HUT adjacent to invasive mammary carcinomas (HUT-IMC), and in neoplastic cells of IMC specimens.

Only nuclear staining was considered in the evaluation of ER, PR, p53, p63, and KI-67. For p21^WAF1^, both nuclear and cytoplasmatic staining were considered positive [35]. We also evaluated ER expression in normal lobules of ARM and MCC specimens, and in adjacent normal lobules of IMC. Two to four lobular units adjacent to HUT areas were evaluated in each case.

Cases were classified as ER, PR, and p53 positive when more than 10% of cells exhibited positive nuclear staining [7,41,42]. The Ki-67 labeling index was obtained by the percentage of neoplastic and HUT cells showing nuclear staining. The tumors and HUT were grouped in three categories: < 10%, low proliferative index; 10–25%, intermediate proliferative index; and > 25%, high proliferative index [43]. We considered p63 positive cases when at least 10% of myoepithelial cells exhibited positive nuclear staining [32]. Cases were classified as p21^WAF1 ^positive when more than 2% of cells exhibited positive nuclear staining [35].

## Abbreviations

HUT- hyperplasia of usual type

ER-estrogen receptor

PR- progesterone receptor

Ki-67- KI-67 protein

p53- p53 protein

TP53- p53 gene

p21^WAF1^- protein p21

p63- protein p63

MCC- mammaplasty contralateral to breast cancer

IMC- invasive mammary carcinomas

ARM- aesthetic reduction mammaplasty

HUT-ARM- hyperplasia of usual type in aesthetic reduction mammaplasty

HUT-MCC in mammaplasty contralateral to breast cancer

HUT-IMC in invasive mammary carcinomas

WHO- world health organization

DCIS- ductal carcinoma in situ

ADH- atypical ductal hyperplasia

G1- cell cycle phase gap 1

## Authors' contributions

LSAT: obtained the samples, carried out the histopathological and immunohistochemical analysis and wrote the first draft of manuscript. GFSR: carried out the histology and immunohistochemistry. HG: conceived and designed the study, confirmed the histopathological and immunohistochemical analysis and provided expert input for writing and supervised the study.

All authors have read and approved the final manuscript.
